# The subacromial index: a novel radiological parameter associated with rotator cuff tears

**DOI:** 10.1016/j.jseint.2026.101742

**Published:** 2026-05-20

**Authors:** Çağrı Üner, Sancar Serbest

**Affiliations:** a Department of Orthopedics and Traumatology, Yozgat City Hospital, Yozgat, Türkiye; b Department of Orthopaedics and Traumatology, Kırıkkale University Faculty of Medicine, Kırıkkale, Türkiye

**Keywords:** Rotator cuff tear, Lateral acromial angle, Acromial index, Critical shoulder angle, Subacromial index, Magnetic resonance imaging

## Abstract

**Background:**

Previous studies have demonstrated associations between radiological parameters such as the acromial index (AI), critical shoulder angle, and lateral acromial angle and rotator cuff tears (RCTs). The aim of this study was to evaluate the relationship between RCTs and a novel measurement method termed the subacromial index (SAI), which has not been previously described in the literature, and to compare this parameter with established radiological measurements.

**Methods:**

This retrospective study evaluated 132 patients who underwent shoulder magnetic resonance imaging between January 2017 and March 2021. The study population was categorized into 2 groups according to the presence or absence of RCTs. Demographic variables and radiological parameters, including age, sex, AI, critical shoulder angle, lateral acromial angle, SAI, and subacromiohumeral distance (SHD), were assessed. Receiver-operating characteristic curve analysis was used to determine cutoff values, and multivariable as well as stratified logistic regression analyses were performed to identify factors associated with RCTs.

**Results:**

Patients with RCTs were older and more frequently female. In the RCT group, AI and critical shoulder angle values were higher, whereas SAI and SHD values were significantly lower. Receiver-operating characteristic analysis demonstrated that older age, female sex, increased AI and critical shoulder angle, and decreased SAI and SHD were associated with RCTs. In the multivariable analysis including demographic variables, age and AI were identified as the strongest factors associated with RCTs. In the stratified analysis excluding demographic variables, the SAI remained significantly associated with RCTs.

**Conclusion:**

The SAI, which simultaneously reflects acromial length and inclination, is significantly associated with RCTs. Although age and AI remain the strongest factors overall, the SAI provides additional anatomical information regarding RCT risk independent of demographic factors.

The rotator cuff muscles constitute a major component of glenohumeral joint function, and rotator cuff tears (RCTs) adversely affect patients' quality of life.^11^ The limited understanding of the etiopathogenesis underlying glenohumeral joint and rotator cuff pathologies responsible for shoulder pain has led to questioning existing clinical assessment approaches and to the development of new evaluation models.^9^

The etiology of RCTs is multifactorial and includes intrinsic factors such as age-related tendon degeneration and vascular changes, extrinsic causes such as mechanical factors related to acromial morphology and the subacromial space, as well as traumatic factors.^2^ Within this context, several radiological parameters related to scapular morphology—namely the acromial index (AI), critical shoulder angle (CSA), and lateral acromial angle (LAA)—have been investigated for their association with degenerative RCTs. Previous studies have evaluated the measurement reliability of these parameters and their individual predictive or discriminative ability in identifying the presence of RCTs.^15^^,^^19^

In this study, a ratio was calculated by dividing the distance between the inferolateral aspect of the acromion and the most lateral point of the humeral head by the distance between the most lateral point of the humeral head and a line joining the superior and inferior margins of the glenoid. This measurement was termed the “subacromial index” (Fig. 1*A*). The main aim of the study was to determine whether this newly defined index, which has not been previously reported in the literature, is capable of predicting the presence of RCTs and to assess its potential advantage over the 3 conventional radiological measurement methods described above.

**Figure 1:**
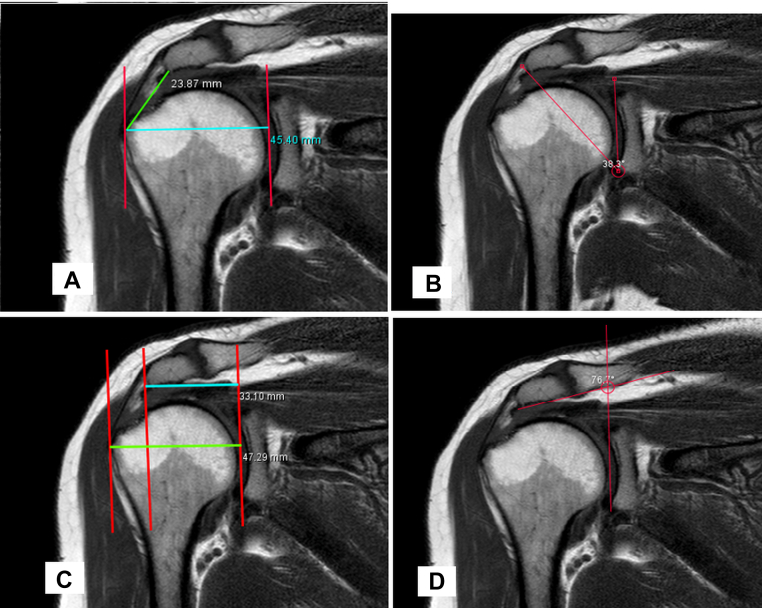
Schematic illustration of radiological measurements used in the study. (**A**) Subacromial index (SAI) defined as the ratio of the subacromiohumeral distance to the humeroglenoidal distance.(**B**) Critical shoulder angle (CSA) measured between a line connecting the superior and inferior margins of the glenoid and a line drawn from the inferior glenoid margin to the most lateral point of the acromion. (**C**) Acromial index (AI) defined as the ratio of the distance from the glenoid plane to the lateral border of the acromion to the distance from the glenoid plane to the most lateral point of the humeral head. (**D**) Lateral acromial angle (LAA) measured between a line parallel to the inferior surface of the acromion and a line representing the glenoid surface.

## Materials and methods

### Ethical approval

Approval for this study was granted by the Local Non-Interventional Research Ethics Committee (May 28, 2021; approval no: 2021.05.10). All procedures were carried out in accordance with the ethical standards of the Declaration of Helsinki.

### Patients

This retrospective analysis reviewed shoulder magnetic resonance imaging examinations of 500 patients performed between January 2017 and March 2021. The eligibility criteria for inclusion were as follows:

• Age ranging from 35 to 60 years
• Absence of a prior history of trauma
• No history of chronic degenerative or inflammatory disease
• Absence of tendinosis or partial-thickness tears on magnetic resonance imaging (MRI)
  A total of 132 patients who met these criteria were included in the study.

Patients were divided into 2 groups, and the data of these groups were compared as follows:

• RCT (−) group: asymptomatic patients without RCTs, who had no shoulder pain or functional complaints and underwent shoulder MRI for reasons unrelated to rotator cuff pathology (n = 59)
• RCT (+) group: patients diagnosed with RCTs on MRI (n = 73)

In addition, patients were stratified according to sex (female and male), and comparisons were performed between these groups.

### Imaging protocol and measurements

All magnetic resonance examinations were carried out using a 1.5-Tesla MRI system (Philips Medical Systems, Achieva, Release 3.2, Level 2013-10-21, the Netherlands). Shoulder magnetic resonance (MR) images were acquired with patients positioned supine on the examination table, while the humerus was maintained in a neutral rotational alignment. All radiological parameters described below, including angular and ratio-based measurements, were obtained directly from these MR images.

### Subacromial index

The subacromial index (SAI) was calculated as a ratio incorporating 2 linear measurements. The numerator was defined as the distance between the inferolateral aspect of the acromion and the most lateral point of the humeral head, referred to as the subacromiohumeral distance (SHD). The denominator represented the distance between the most lateral point of the humeral head and a line connecting the inferior-most and superior-most points of the glenoid cavity, defined as the humeroglenoidal distance. The ratio of these 2 measurements was defined as the SAI (Fig. 1*A*).

### Critical shoulder angle

The CSA was measured according to the previously described method. This angle was defined by the intersection of 2 reference lines: the first line connecting the inferior-most and superior-most points of the lateral margin of the glenoid cavity, and the second line extending from the inferior lateral point of the glenoid cavity to the most lateral point of the acromion (Fig. 1*B*). This measurement reflects the combined influence of glenoid inclination and lateral acromial extension on shoulder biomechanics.^16^

### Acromial index

The AI was calculated as a ratio reflecting the relative lateral extension of the acromion. Specifically, it was defined as the ratio of the distance between the glenoid plane and the lateral border of the acromion (blue line) to the distance between the glenoid plane and the most lateral aspect of the proximal humerus (green line) (Fig. 1*C*).^17^ This parameter was used to quantify lateral acromial coverage in relation to humeral head position.

### Lateral acromial angle

The LAA was measured using 2 reference lines. The first line was drawn parallel to the inferior surface of the acromion, while the second line represented the glenoid surface and was oriented parallel to the superior and inferior osseous margins of the glenoid cavity. The angle formed at the intersection of these 2 lines was defined as the LAA (Fig. 1*D*).^3^

### Statistical analysis

All statistical evaluations were carried out using the Statistical Package for the Social Sciences software (SPSS, version 20.0; IBM Corp, Armonk, NY, USA). Categorical variables were compared between groups using the Pearson chi-square test. For continuous variables, data with a normal distribution were analyzed using the independent-samples *t*-test, whereas variables not meeting parametric assumptions were assessed using the Mann–Whitney *U* test. A *P* value of less than .05 was considered statistically significant for all analyses.

Receiver-operating characteristic (ROC) curve analysis was used to examine the ability of the studied parameters to discriminate between patients with and without RCTs. Cutoff values were identified based on sensitivity and specificity. To determine variables independently associated with the presence of RCTs, multivariable logistic regression analysis was performed. In the initial regression model, age, sex, and all anatomical parameters were included simultaneously. In a second model, demographic variables (age and sex) were excluded to evaluate the independent contribution of anatomical parameters after controlling for demographic effects. This stratified modeling approach was used to assess the additional predictive value provided by anatomical measurements in relation to RCT risk (*P* < .05).

The magnitude and direction of associations between patient-related variables and the risk of RCTs were expressed as odds ratios (ORs) together with their corresponding 95% confidence intervals (CIs).

To evaluate the reproducibility of SAI measurements, assessments were performed by 2 independent radiology specialists and 1 orthopedic surgeon, each of whom had more than 5 years of experience in musculoskeletal imaging. Measurements were conducted on magnetic resonance images of 30 randomly selected patients. Intraobserver reliability was assessed by repeating the measurements on the same images after an interval of 2 weeks. All measurements were obtained in a blinded fashion with respect to prior results. Interobserver and intraobserver reliability were analyzed using a 2-way mixed-effects model to calculate the intraclass correlation coefficient (ICC). An ICC value greater than 0.75 was considered indicative of good measurement reliability.

## Results

A total of 132 patients were included in the analysis. Full-thickness RCTs were identified on magnetic resonance imaging in 73 patients (55.3%), whereas 59 patients (44.7%) demonstrated an intact rotator cuff. The study population consisted of 72 female patients (54.5%) and 60 male patients (45.5%), and a statistically significant difference in sex distribution was observed between groups (χ^2^ = 8.275, *P* = .004). The median age was significantly higher in the RCT-positive group compared with the tear-negative group (52 years [range, 38–58] vs. 44 years [range, 35–60], respectively; Z = −6.056, *P* < .001). These findings indicate that RCTs occurred more frequently among female patients and in older individuals (Table I).

**Table I:** Comparison of demographic characteristics and radiological measurements between patients with and without rotator cuff tears.

Variable	RCT (−)	RCT (+)	t/Z/X^2^	*P* value
	Mean ± SD/median (min-max) N (%)	Mean ± SD/median (min-max) N (%)		
Age	44 (35-60)	52 (38-58)	−6.056†	**<.001**
Sex				
Male	35 (26.5%)	25 (18.9%)	8.275‡	**.004**
Female	24 (18.2%)	48 (36.4%)		
Acromial index	0.56 ± 0.08	0.60 ± 0.08	−2.670∗	**.009**
Critical shoulder angle	29.30 ± 4.56	31.57 ± 4.76	−2.775∗	**.006**
Lateral acromial angle	82.60 ± 6.38	80.66 ± 6.06	1.790∗	.076
Subacromial index	0.61 ± 0.08	0.56 ± 0.10	3.033∗	**.003**
Humeroglenoidal distance	45.01 ± 3.84	43.52 ± 3.86	2.214∗	**.029**
Subacromiohumeral distance	27.59 ± 4.64	24.85 ± 5.04	3.226∗	**.002**

*SD*, standard deviation; *N*, number of patients; *min*, minimum; *max*, maximum; *RCT*, rotator cuff tear.

Bold values indicate statistical significance (*P* < .05)

∗ t value, *independent samples t-test*.

† Z value, *Mann-Whitney U test*.

‡ X^2^ value, *Pearson’s chi-square test*, *P < .05.*

**Figure 2:**
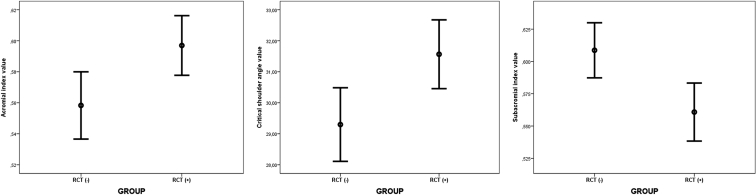
Comparison of selected radiological parameters between patients with and without rotator cuff tears. Box plots illustrate the distribution and differences in acromial index, critical shoulder angle, and subacromial index values between the RCT-positive and RCT-negative groups. *RCT*, rotator cuff tear.

Comparative analysis between groups revealed statistically significant differences in multiple anatomical parameters. AI (t = −2.670, *P* = .009), CSA (t = −2.775, *P* = .006), SAI (t = 3.033, *P* = .003), humeroglenoidal distance (t = 2.214, *P* = .029), and SHD (t = 3.226, *P* = .002) all differed significantly between patients with and without RCTs. Patients in the RCT-positive group exhibited higher AI and CSA values, whereas humeroglenoidal and SHD values were significantly lower. Although LAA values did not differ significantly between groups (*P* = .076), a tendency toward lower values was observed in patients with RCTs (Table I, Fig. 2).

Sex-based comparisons demonstrated significant differences with respect to age (Z = −3.158, *P* = .002), humeroglenoidal distance (t = 9.656, *P* < .001), and SHD (t = 4.393, *P* < .001). Female patients were older and showed lower humeroglenoidal and SHD values compared with male patients (Table II).

**Table II:** Comparison of demographic characteristics and radiological measurements between male and female patients.

Variable	Male	Female	t/Z/X^2^	*P* value
	Mean ± SD/median (min-max) N (%)	Mean ± SD/median (min-max) N (%)		
Age	44 (35-60)	52 (37-58)	−3.158†	**.002**
Rotator cuff tears			8.275‡	**.004**
No	35 (26.5%)	24 (18.2%)		
Yes	25 (18.9%)	48 (36.4%)		
Acromial index	0.58 ± 0.08	0.58 ± 0.09	−0.374∗	.709
Critical shoulder angle	30.30 ± 4.74	30.76 ± 4.85	−0.555∗	.580
Lateral acromial angle	81.02 ± 6.78	81.94 ± 5.78	−0.840∗	.402
Subacromial index	0.59 ± 0.09	0.58 ± 0.10	0.843∗	.401
Humeroglenoidal distance	46.95 ± 2.91	41.89 ± 3.06	9.656∗	**<.001**
Subacromiohumeral distance	28.05 ± 4.68	24.43 ± 4.75	4.393∗	**<.001**

*SD*, standard deviation; *N*, number of patients; *min*, minimum; *max*, maximum.

Bold values indicate statistical significance (*P* < .05).

∗ t value, *independent samples t-test*.

† Z value, *Mann-Whitney U test*.

‡ X^2^ value, *Pearson's chi-square test*, *P < .05.*

Correlation analysis demonstrated positive associations between the presence of RCTs and age (r = 0.529, *P* < .001), sex (r = 0.250, *P* = .004), AI (r = 0.228, *P* = .008), and CSA (r = 0.240, *P* = .006). In contrast, negative correlations were identified between RCT probability and both SAI (r = −0.258, *P* = .003) and SHD (r = −0.262, *P* = .002). These findings suggest that increasing age, female sex, and higher AI and CSA values are associated with a greater likelihood of RCTs, whereas reduced SAI and SHD values are likewise associated with increased risk.

Additional correlation analysis revealed a positive association between SAI and LAA (r = 0.363, *P* < .001). Conversely, SAI demonstrated negative correlations with both AI (r = −0.619, *P* < .001) and CSA (r = −0.618, *P* < .001). These relationships indicate that lower SAI values tend to coexist with lower LAA values and higher AI and CSA measurements, a combination associated with an increased likelihood of RCTs.

ROC curve analysis showed that age greater than 47 years (area under the curve [AUC] = 0.807, *P* < .001; sensitivity 77%, specificity 70%), female sex (AUC = 0.625, *P* = .013; sensitivity 66%, specificity 60%), AI greater than 0.58 (AUC = 0.632, *P* = .009; sensitivity 60%, specificity 63%), and CSA greater than 30.55° (AUC = 0.639, *P* = .006; sensitivity 59%, specificity 63%) demonstrated moderate diagnostic performance for predicting RCTs.

**Figure 3:**
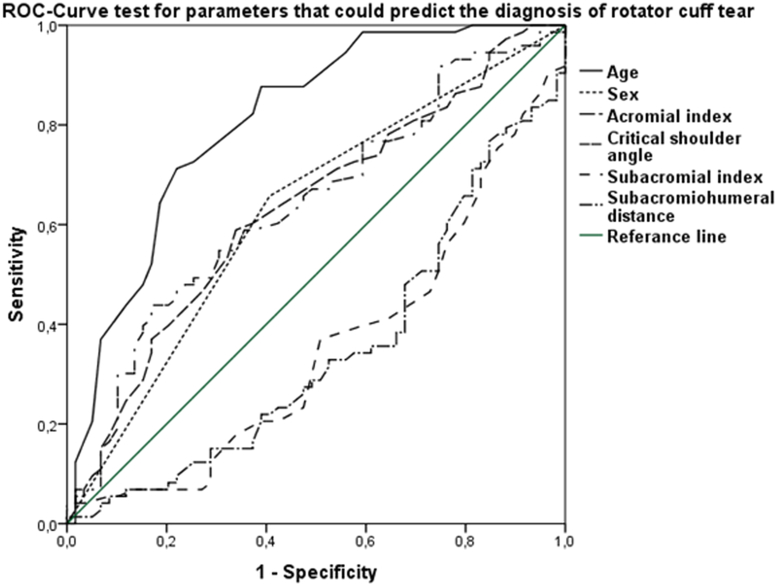
Receiver-operating characteristic (ROC) curves of radiological and demographic parameters associated with rotator cuff tears. ROC curves illustrate the discriminative performance of age, sex, acromial index, critical shoulder angle, subacromial index, and subacromiohumeral distance for the diagnosis of rotator cuff tears. *ROC*, receiver-operating characteristic.

In contrast, the area under the ROC curve for SAI and SHD was below 0.5 (AUC = 0.350 and AUC = 0.348, respectively). This finding reflects the inverse biological relationship between these parameters and the presence of RCTs, indicating that ROC analysis for these measurements primarily represents the direction of association rather than discriminatory ability (Table III, Fig. 3).

**Table III:** Receiver-operating characteristic curve analysis of parameters associated with rotator cuff tears.

Variable	ROC curve analysis of parameters associated with rotator cuff tear				
	AUC	*P* value	Cutoff value	Sensitivity	Specificity
Age	0.807	**<.001**	>47	77%	70%
Sex	0.625	**.013**	Female	66%	60%
Acromial index	0.632	**.009**	>0.58	60%	63%
Critical shoulder angle	0.639	**.006**	>30.55	59%	63%
Subacromial index	0.350	**.003**	<0.58	60%	64%
Subacromiohumeral distance	0.348	**.003**	<25.75	64%	66%

*ROC*, receiver-operating characteristic; *AUC*, area under the curve.

Bold values indicate statistical significance (*P* < .05).

Multivariate logistic regression analysis identified age (B = 0.216, Wald = 29.188, *P* < .001) and AI (B = 6.720, Wald = 5.993, *P* = .014) as the strongest independent predictors of RCTs. Sex was also identified as an independent risk factor (OR = 2.80, *P* = .004).

Given that age and sex are known to be strong determinants, a stratified analysis was performed to evaluate the independent contribution of anatomical parameters beyond these demographic factors. In this analysis, age and sex were deliberately excluded from the model, and only anatomical parameters were assessed. Within this framework, the SAI was found to be significantly associated with the diagnosis of RCTs (B = −6.025, Wald = 8.138, *P* = .004). The overall model accuracy was calculated as 62.9%. This finding suggests that the SAI may provide additional anatomical information regarding RCT risk independent of demographic factors (Table IV).

**Table IV:** Stratified logistic regression analysis evaluating the independent association between the subacromial index and rotator cuff tears after exclusion of demographic variables.

ROC curve analysis for predicting the diagnosis of RCT					
Variable	AUC	*P* value	Cutoff value	Sensitivity	Specificity
Acromial index	0.632	.009	>0.58	60%	63%
Critical shoulder angle	0.639	.006	>30.55	59%	63%
Subacromial index	0.350	.003	<0.58	60%	64%

Stratified logistic regression analysis of subacromial index associated with rotator cuff tear					
Variable	Expected				
Subacromial index			Healthy	Tear	Percentage
	Observed	Healthy	25	34	42.4%
		Tear	15	58	79.5%
	Overall accuracy				62.9%

Variable			B	Wald	*P* value
Subacromial index			−6.025	8.138	0.004

*RCT*, rotator cuff tear; *ROC*, receiver-operating characteristic; *AUC*, area under the curve.

**Figure 4:**
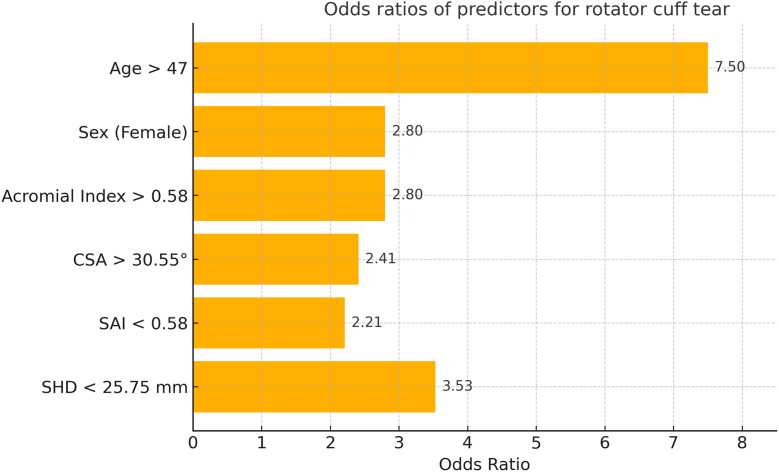
Odds ratio analysis of factors associated with rotator cuff tears. Forest plot demonstrates odds ratios and 95% confidence intervals for demographic and radiological variables significantly associated with rotator cuff tear risk. *CSA*, critical shoulder angle; *SAI*, subacromial index; *SHD*, subacromiohumeral distance.

According to OR analysis, the risk of RCTs increased approximately 7.5-fold in patients older than 47 years (OR = 7.50, 95% CI: 3.45–16.30; *P* < .001). Female sex (OR = 2.80; 95% CI: 1.38–5.69; *P* = .004), AI >0.58 (OR = 2.80; 95% CI: 1.37–5.70; *P* = .005), CSA >30.55° (OR = 2.41; 95% CI: 1.19–4.88; *P* = .014), and SAI <0.58 (OR = 2.21; 95% CI: 1.10–4.45; *P* = .026) were also associated with a significantly increased risk of RCTs. A SHD <25.75 mm was associated with more than a threefold increase in risk (OR = 3.53; 95% CI: 1.71–7.25; *P* = .001) (Table V, Fig. 4).

**Table V:** Odds ratio analysis of demographic and radiological parameters associated with rotator cuff tear risk.

Variable	Risk of the rotator cuff tear					95% CI	
	Cutoff value	RCT (−)	RCT (+)	OR	*P* value	Lower	Upper
Age	≤47	41 (31.1%)	17 (12.9%)	7.50	<.001	3.45	16.30
	>47	18 (13.6%)	56 (42.4%)				
Sex	Male	35 (26.5%)	25 (18.9%)	2.80	.004	1.38	5.69
	Female	24 (18.2%)	48 (36.4%)				
Acromial index	≤0.58	39 (29.5%)	30 (22.7%)	2.80	.005	1.37	5.70
	>0.58	20 (15.2%)	43 (32.6%)				
Critical shoulder angle	≤30.55	37 (28.0%)	30 (22.7%)	2.41	.014	1.19	4.88
	>30.55	22 (16.7%)	43 (32.6%)				
Subacromial index	≤0.58	24 (18.2%)	44 (33.3%)	2.21	.026	1.10	4.45
	>0.58	35 (26.5%)	29 (22.0%)				
Subacromiohumeral distance	≤25.75	20 (15.2%)	47 (35.6%)	3.53	.001	1.71	7.25
	>25.75	39 (29.5%)	26 (19.7%)				

*RCT*, rotator cuff tear; *OR*, odds ratio; *CI*, confidence interval.

Reliability analysis performed to assess the reproducibility of SAI measurements demonstrated an interobserver ICC of 0.82 (95% CI: 0.68–0.91) based on measurements performed by 1 orthopedic surgeon and 1 radiology specialist on MR images of 30 randomly selected patients, indicating good interobserver agreement. Intraobserver reliability analysis revealed an ICC of 0.89 (95% CI: 0.79–0.95), indicating excellent agreement. These findings confirm that SAI measurement is reproducible and reliable.

The anatomical parameters evaluated in this study were not intended to form a single composite clinical scale but were analyzed to determine their individual associations with RCTs. Therefore, internal consistency among parameters or the development of a combined scoring system was not pursued.

## Discussion

A considerable proportion of RCTs is thought to arise from subacromial impingement, particularly due to mechanical compression associated with variations in acromial morphology.^14^ Nevertheless, the association between individual scapular anatomical characteristics and the occurrence of RCTs has not yet been fully clarified. Following Bigliani's original classification of acromial types, several additional radiological parameters, including acromial slope, have been proposed over time.^4^^,^^8^ Despite documented differences in acromial shape and inclination, the mechanisms explaining why RCTs develop in some individuals but not in others remain incompletely understood.

The AI provides a quantitative assessment of acromial morphology by measuring the degree of lateral acromial extension. However, previous investigations evaluating the predictive value of the AI for RCTs have reported inconsistent findings.^5^^,^^12^^,^^17^^,^^21^ Nyffeler et al proposed that increased lateral extension of the acromion modifies the direction of the deltoid muscle force vector, thereby contributing to progressive rotator cuff degeneration.^17^ Similarly, Kim et al observed that elevated AI values were more prevalent in large and massive RCTs, suggesting that a higher AI may represent a risk factor for advanced tear severity.^7^ In the present analysis, AI emerged as the second most strongly associated variable with RCTs after age in multivariate logistic regression analysis (B = 6.720, Wald = 5.993, *P* = .014, OR = 2.80). These results reinforce the importance of the AI as a relevant anatomical factor in RCT development.

The CSA, first described by Moor et al, represents a biomechanical parameter that influences shoulder abduction mechanics, glenoid loading, and intra-articular shear forces. This measurement is considered sensitive and may vary according to scapular orientation and acromial positioning.^22^ İncesoy et al reported a significant association between CSA values measured on magnetic resonance imaging and full-thickness RCTs, with CSA measurements comparable to those obtained in the current study.^6^ Consistent with these findings, our results demonstrated a statistically significant difference in CSA values between patients with and without RCTs, with higher CSA values observed in the tear group. This supports the relevance of CSA in assessing RCT risk.

The relationship between LAA and RCTs has also been widely investigated. Banas et al, who initially defined the LAA, demonstrated that this parameter could be reliably measured on magnetic resonance images and reported that reduced LAA values were associated with a markedly increased likelihood of RCTs.^16^ Likewise, Singleton et al identified lower LAA values in patients with RCTs compared with control subjects using radiographic and computed tomography imaging.^20^ Conversely, Maalouly et al found no significant differences in acromial measurements, including LAA, between patients with and without RCTs and reported no sex-related influence on these parameters.^10^

In the present study, although no statistically significant difference in LAA was detected between groups, lower LAA values were observed among patients with RCTs. This tendency suggests that acromial inclination may contribute to RCT development in certain individuals. Accordingly, LAA may represent a predisposing factor for RCTs in selected cases; however, additional studies are required before this association can be generalized across broader populations.

The acromiohumeral distance (AHD) is defined as the minimal distance between the undersurface of the acromion and the humeral head and is widely regarded as the most direct radiological marker of subacromial space reduction. Saupe et al demonstrated a significant inverse association between reduced AHD values and multiple characteristics of RCTs, including their presence, anatomical location, tear size, and degree of fatty infiltration.^18^ These observations indicate that AHD is a meaningful parameter in the evaluation of RCT risk. Nevertheless, the direction of causality between AHD narrowing and RCT development has not been clearly established. It remains uncertain whether a decreased AHD predisposes to RCTs by increasing mechanical compression within the subacromial space or whether it occurs secondary to superior migration of the humeral head following tendon rupture.

Additional insight into this issue was provided by a systematic review conducted by Alharairi et al in 2025, which reported that a reduced AHD is not universally present in patients with RCTs.^1^ Some individuals were shown to have substantial tears despite preservation of a normal AHD. This finding supports the concept that AHD narrowing alone does not fully explain RCT pathogenesis. Rather, a decreased AHD may function both as a contributing factor—by promoting mechanical impingement—and as a secondary manifestation resulting from altered humeral head positioning after tear formation. Further investigations are required to better delineate the underlying cause–effect relationship.

The morphology of the subacromial space plays a critical role in the development of RCTs. However, each of the currently used anatomical parameters reflects only a specific component of the subacromial space. The AI evaluates lateral acromial extension, whereas the AHD assesses the vertical component of the subacromial space.

Individuals with the same AI may have different AHDs, and conversely, individuals with similar AHDs may exhibit differences in AI. These observations suggest that RCT risk may not be adequately explained by any single parameter alone.

The SHD is defined as the distance between the inferolateral border of the acromion and the most lateral point of the humeral head. SHD simultaneously reflects lateral acromial extension, inferior acromial inclination, and changes in AHD. Increased lateral extension of the acromion, increased inferior inclination, or a decrease in AHD may all result in a reduced SHD. Consequently, SHD serves as a comprehensive indicator incorporating both the vertical and lateral components of the subacromial space.

The SAI is defined as the ratio of the SHD to the humeroglenoidal distance. This ratio normalizes the subacromial space by eliminating interindividual differences in humeral and glenoidal dimensions. SAI may help explain why individuals with similar acromial indices but different subacromial space widths, or those with comparable subacromial spaces but differing acromial extensions, demonstrate varying risks of RCTs. The significant association between SAI and RCTs observed in the present study suggests that a multidimensional assessment of subacromial space morphology may be clinically relevant.

In the present study, SHD values were significantly lower in the RCT group compared with the control group. ROC analysis demonstrated that an SHD threshold of <25.75 mm could predict RCT diagnosis. The area under the ROC curve for SHD (AUC = 0.680) indicates that this parameter has good predictive value for RCT risk.

Similarly, SAI values were significantly lower in the RCT group. ROC analysis demonstrated that an SAI threshold of <0.58 could predict RCT diagnosis. However, the area under the ROC curve for SAI (AUC = 0.350) appeared lower compared with other parameters.

The low AUC value observed for the SAI is attributable to the inverse biological effect of this parameter. In other words, an increase in SAI is associated with a reduced risk of RCTs, whereas a decrease in SAI is associated with an increased risk. This inverse relationship does not diminish the clinical significance of the SAI; rather, it supports the association between lower SAI values and an increased likelihood of RCTs.

The present study demonstrated a significant association between SAI and the presence of RCTs. The increased likelihood of RCTs with decreasing SAI values suggests that this parameter reflects both subacromial space narrowing and the biomechanical effects of acromial morphology.

Although age and AI emerged as the strongest independent variables in multivariate logistic regression analysis, the finding that SAI remained significantly associated with RCT risk independent of demographic factors indicates that this parameter may serve a complementary role in anatomical assessment.

Overall, none of these measurements alone appears sufficient to fully predict RCTs. This supports the concept that RCTs are truly multifactorial, with contributing roles played not only by anatomical parameters but also by age, sex, trauma history, activity level, and intrinsic tendon quality. The clinical value of the SAI becomes apparent when it is considered as part of a comprehensive evaluation alongside other anatomical and demographic factors.

The applicability of the SAI, which was defined based on MRI in the present study, to plain radiographs represents an important topic of clinical discussion. Although the accessibility of radiographic parameters such as the AI and CSA on plain radiographs has been investigated in the literature, the applicability of the SAI defined in this study to radiographs remains unclear. While MRI allows direct visualization of RCTs, plain radiographs are more commonly used in clinical practice due to their lower cost and greater accessibility. In the study by Schiefer et al, both the AI and CSA demonstrated high intra- and interobserver reliability when measured on both radiographs and MRI, with strong correlation between the 2 imaging modalities.^19^ On the other hand, Mirzayan et al reported that measurements of the AHD may differ between radiographs and MRI and that these modalities should not be used interchangeably.^13^ Therefore, although a significant association between the SAI and RCTs was demonstrated in the present study, the reliability of this newly defined parameter on plain radiographs and its potential to reduce the need for advanced imaging should be investigated in future studies.

However, this study has several methodological limitations. First, the study has a retrospective design and was conducted at a single center. Second, due to the inverse relationship between the SAI and RCTs, recoding of the variable direction for ROC analysis could have been considered. However, reaccess to the raw patient data was not possible, and; therefore, the ROC analyses could not be restructured. This may have led to an underestimation of the discriminative performance of the SAI. Third, the heterogeneity of MR imaging indications among patients in the control group may have introduced potential selection bias. Finally, the restriction of the study population to patients aged between 35 and 60 years limits the generalizability of the findings to older age groups.

## Conclusion

The etiology of RCTs is complex, and both demographic factors and anatomical parameters have been shown to play important roles. However, further studies are required to better understand the individual clinical value of these parameters and their interactions.

In particular, large-scale prospective studies are needed to determine whether anatomical parameters can predict the development of RCTs in asymptomatic individuals. Longitudinal studies are required to clarify the cause–effect relationship between decreased AHD and RCTs. In addition, the newly defined SAI should be validated across different centers and populations. The potential utility of anatomical parameters in predicting treatment selection and prognosis in RCTs should also be investigated.

Such studies may contribute to improvements in the diagnosis, treatment, and prognosis of RCTs.

## Disclaimers:

Funding: No funding was disclosed by the authors.

Conflicts of interest: The authors, their immediate families, and any research foundations with which they are affiliated have not received any financial payments or other benefits from any commercial entity related to the subject of this article.

## References

[1] Alharairi S., Vincent J. (2025). Exploring the level of association between rotator cuff tears and acromiohumeral distance: a systematic review. JSES Rev Rep Tech.

[2] Balke M., Liem D., Greshake O., Hoeher J., Bouillon B., Banerjee M. (2016). Differences in acromial morphology of shoulders in patients with degenerative and traumatic supraspinatus tendon tears. Knee Surg Sports Traumatol Arthrosc.

[3] Banas M.P., Miller R.J., Totterman S. (1995). Relationship between the lateral acromion angle and rotator cuff disease. J Shoulder Elbow Surg.

[4] Bigliani L.U., Morrison D.S., April E.W. (1986). The morphology of the acromion and its relationship to rotator cuff tears. Orthop Trans.

[5] Hamid N., Omid R., Yamaguchi K., Steger-May K., Stobbs G., Keener J.D. (2012). Relationship of radiographic acromial characteristics and rotator cuff disease: a prospective investigation of clinical, radiographic, and sonographic findings. J Shoulder Elbow Surg.

[6] İncesoy M.A., Yıldız K.I., Türk Ö.I., Akıncı Ş., Turgut E., Aycan O.E. (2021). The critical shoulder angle, the acromial index, the glenoid version angle and the acromial angulation are associated with rotator cuff tears. Knee Surg Sports Traumatol Arthrosc.

[7] Kim J.R., Ryu K.J., Hong I.T., Kim B.K., Kim J.H. (2012). Can a high acromion index predict rotator cuff tears?. Int Orthop.

[8] Kitay G.S., Iannotti J.P., Williams G.R., Haygood T., Kneeland B.J., Berlin J. (1995). Roentgenographic assessment of acromial morphologic condition in rotator cuff impingement syndrome. J Shoulder Elbow Surg.

[9] Lewis J.S. (2009). Rotator cuff tendinopathy/subacromial impingement syndrome: is it time for a new method of assessment?. Br J Sports Med.

[10] Maalouly J., Tawk A., Aouad D., Abdallah A., Darwiche M., Abboud G. (2020). Association of acromial morphological parameters and rotator cuff tears, and evaluation of the influence of age and gender on the parameters and impact on cuff tears: a study on a Middle Eastern population. Asia Pac J Sports Med Arthrosc Rehabil Technol.

[11] MacDermid J.C., Ramos J., Drosdowech D., Faber K., Patterson S. (2004). The impact of rotator cuff pathology on isometric and isokinetic strength, function, and quality of life. J Shoulder Elbow Surg.

[12] Mahmoud M.A., Azab E.A., Gamal Eldeen N.M. (2025). Degenerative rotator cuff tears in correlation with different anatomic shoulder parameters on MRI. Egypt J Radiol Nucl Med.

[13] Mirzayan R., Donohoe S., Batech M., Suh B.D., Acevedo D.C., Singh A. (2020). Is there a difference in the acromiohumeral distances measured on radiographic and magnetic resonance images of the same shoulder with a massive rotator cuff tear?. J Shoulder Elbow Surg.

[14] Mombellet M., Samargandi R., Berhouet J. (2025). Reproducibility and relevance of acromial morphology measurements in shoulder pathologies: a critical review of the literature. J Clin Med.

[15] Moor B.K., Bouaicha S., Rothenfluh D.A., Sukthankar A., Gerber C. (2013). Is there an association between the individual anatomy of the scapula and the development of rotator cuff tears or osteoarthritis of the glenohumeral joint?: a radiological study of the critical shoulder angle. Bone Joint J.

[16] Moor B.K., Wieser K., Slankamenac K., Gerber C., Bouaicha S. (2014). Relationship of individual scapular anatomy and degenerative rotator cuff tears. J Shoulder Elbow Surg.

[17] Nyffeler R.W., Werner C.M., Sukthankar A., Schmid M.R., Gerber C. (2006). Association of a large lateral extension of the acromion with rotator cuff tears. J Bone Joint Surg Am.

[18] Saupe N., Pfirrmann C.W., Schmid M.R., Jost B., Werner C.M., Zanetti M. (2006). Association between rotator cuff abnormalities and reduced acromiohumeral distance. AJR Am J Roentgenol.

[19] Schiefer M., Naliato E., Oliveira R., Carmo L.T.D., Fontenelle C.R.D.C. (2023). Motta Filho GDR MRI is a reliable method for measurement of critical shoulder angle and acromial index. Rev Bras Ortop (Sao Paulo).

[20] Singleton N., Agius L., Andrews S. (2017). The acromiohumeral centre edge angle: a new radiographic measurement and its association with rotator cuff pathology. J Orthop Surg (Hong Kong).

[21] Torrens C., Lopez J.M., Puente I., Caceres E. (2007). The influence of the acromial coverage index in rotator cuff tears. J Shoulder Elbow Surg.

[22] Vellingiri K., Ethiraj P., Shanthappa A.H. (2020). Critical shoulder angle and its clinical correlation in shoulder pain. Cureus.

